# S. T. Von Soemmering

**Published:** 1830-05

**Authors:** 


The celebrated S. T. VON SOEMMERING, who maintained so high a rank as an anatomist and physiologist, departed this life at Frankfort, on the 2d of March, in the seventy-sixth year of his age.

